# Modeling Natural Killer Cell Targeted Immunotherapies

**DOI:** 10.3389/fimmu.2017.00370

**Published:** 2017-03-29

**Authors:** Silvia Lopez-Lastra, James P. Di Santo

**Affiliations:** ^1^Innate Immunity Unit, Institut Pasteur, Paris, France; ^2^Inserm U1223, Paris, France; ^3^Université Paris-Sud (Paris-Saclay), Paris, France

**Keywords:** humanized mouse models, innate lymphoid cell, natural killer cells, cancer immunotherapy, natural killer cell immunotherapy

## Abstract

Animal models have extensively contributed to our understanding of human immunobiology and to uncover the underlying pathological mechanisms occurring in the development of diseases. However, mouse models do not reproduce the genetic and molecular complexity inherent in human disease conditions. Human immune system (HIS) mouse models that are susceptible to human pathogens and can recapitulate human hematopoiesis and tumor immunobiology provide one means to bridge the interspecies gap. Natural killer cells are the founding member of the innate lymphoid cell family. They exert a rapid and strong immune response against tumor and pathogen-infected cells. Their antitumor features have long been exploited for therapeutic purposes in the context of cancer. In this review, we detail the development of highly immunodeficient mouse strains and the models currently used in cancer research. We summarize the latest improvements in adoptive natural killer (NK) cell therapies and the development of novel NK cell sources. Finally, we discuss the advantages of HIS mice to study the interactions between human NK cells and human cancers and to develop new therapeutic strategies.

## Introduction

Since the generation of the first inbred mouse strains in the early 20th century, mice have served as model organisms to study mammalian biology. This approach has given birth to some of the most important scientific breakthroughs and discoveries that, in many cases, led to the development of successful treatments for previously untreatable diseases (e.g., acute promyelocytic leukemia) (1). However, *Mus musculus* and *Homo sapiens* have been evolving divergently for 85 million years, adapting to very different environments and undergoing selection for many traits, from the circadian rhythm to our body size (2). Thanks to the genome decoding, we can now appreciate that the one fifth of the genetic divergence between mice and humans is enriched in regions implicated in the immune system, metabolic processes, and stress responses (3). It is, therefore, not surprising that only less than 8% of the cancer studies in animal models reach clinical trials and that more than 80% of these eventually fail when tested in humans (4). The increasing knowledge of the molecular differences between mice and humans should allow us to evaluate the degree in which animal models may be suitable for translational research and when this is not the case, to then search for better systems.

With this aim, mice have been “humanized” by introducing human genes or genomic regions and by transferring human tissues or cells to study various aspects of human biology. The engraftment of human blood cells or blood-forming cells and organs into immunodeficient mice has opened a new era for translational immunology and the improvement of immunotherapies against human cancer and infectious diseases caused by pathogens with exclusive human tropism, such as HIV, HBV, and HCV.

## Developing Human Immune System (HIS) Mice

Since the discovery of the nude athymic mutations in the 1960s, our knowledge of the host immune system and its ability to reject xenografts have led to the development of several mouse strains that permit long-term “take” and function of the human tissue grafts (5). Experiments performed in the 1980s with severe combined immunodeficient (SCID) mice (that lacked functional mouse adaptive lymphocytes due to mutations in the DNA-dependent protein kinase *Prkdc*) showed that these mice could be reconstituted with human peripheral blood mononuclear cells (PBMCs) or hematopoietic stem cells (HSCs) (6, 7). However, some residual adaptive (leakiness) and an essentially intact innate immunity in SCID mice limited the complete reconstitution of all human immune subsets. Moreover, SCID mice failed to engraft human tumor xenografts, thereby limiting the development of preclinical cancer models. An alternative system with analogous immunodeficiency was obtained by mutating the recombinant activating genes (*Rag1, Rag2*) loci that avoided genetic “leakiness” and, in contrast to SCID mice, did not result in host radiosensitivity (8, 9). Additional genetic modifications followed to further the immunodeficiency of host mice in order to promote tolerance to human cells. Two breakthroughs have remarkably boosted the advancement of the field. First, Greiner and colleagues found that the NOD strain supported an enhanced tolerance compared to other strains and, several years later, Takenaka’s team revealed that the molecular basis for this lies in the signal regulatory protein alpha (*Sirpa*) allele polymorphism (10–13). Contrarily to other strains, SIRPα from NOD mice binds to human CD47 ligand triggering a negative signal in mouse macrophages that prevents their phagocytosis (13, 14). This finding prompted the generation of transgenic mice expressing the human or NOD strain *Sirpa* allele thus conferring enhanced human cell engraftment (15–17). The second turning point for achieving a successful xenotransplantation was the common cytokine receptor gamma chain (γ_c_, encoded at *Il2rg*), which leads to complete impairment of natural killer (NK) cell development and dendritic cell (DC) dysfunction (18, 19). Mice carrying *Il2rg* mutations were developed in various genetic backgrounds [NSG or NOG (both NOD *Prkdc*^SCID^*Il2rg^−/−^*) and BRG (Balb/c *Rag2^−/−^Il2rg^−^*^/^*^−^*)] allowing robust, long-lasting *de novo* multilineage development of the HIS, including human thymopoiesis, and are the basis for most of the currently used models (20–23). From that point forward, a number of model variants have been developed to address specific questions or improve particular aspects of immunity, either by genetic manipulation, engraftment of additional human tissues, or exogenous administration of human factors. This is the case of the recently described Balb/c *Rag2^−/−^Il2rg^−/−^Flt3^−/−^* (BRGF) model with specific boost of conventional and plasmacytoid DCs after exogenous Flt3 ligand treatment. This model offers a great platform for screening of immune adjuvants and DC targeting therapies (24).

## Human Cancer Models in “Humanized” Mice

**Figure 1:**
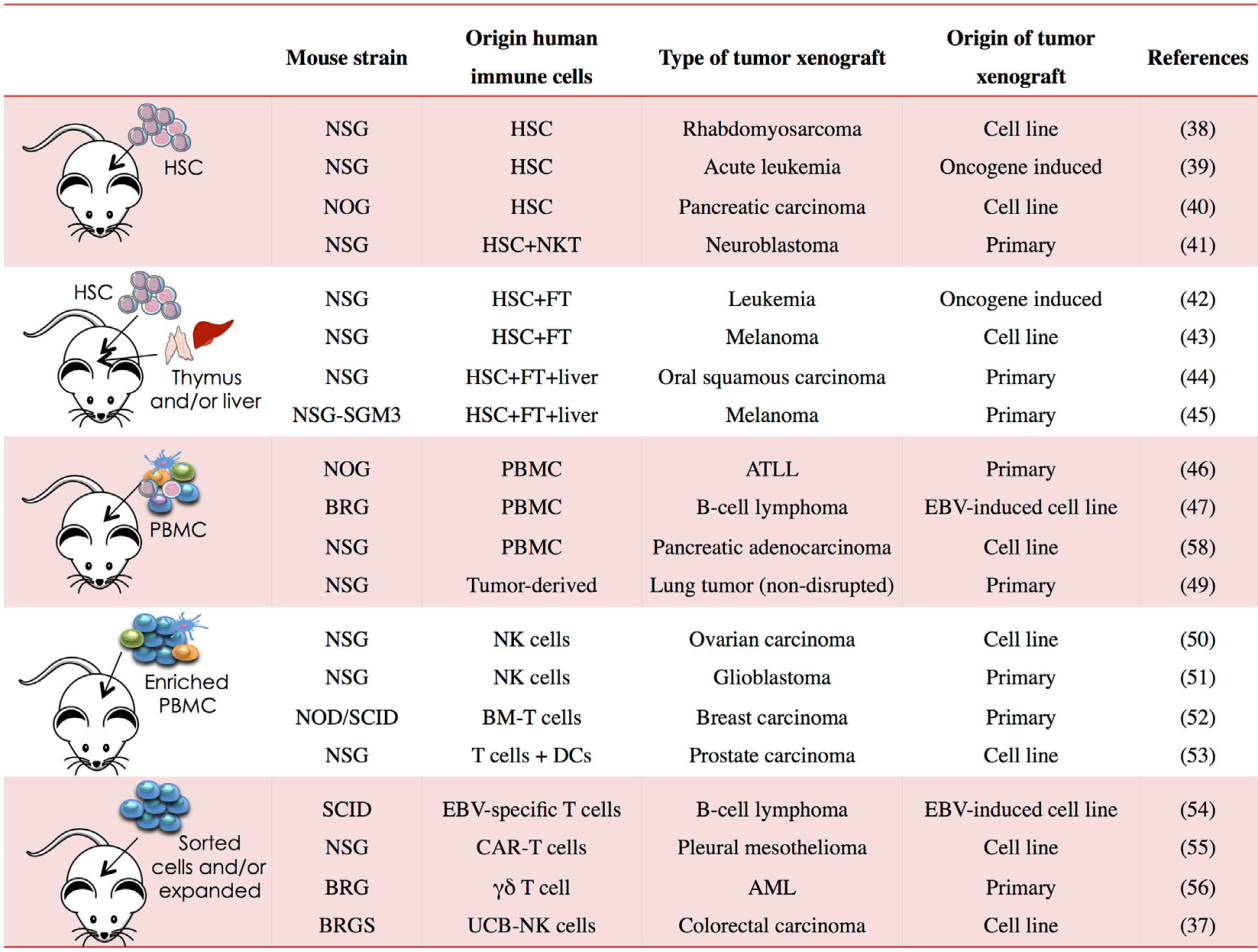
**Human immune system (HIS) mouse models used in cancer research**. PBMC, peripheral blood mononuclear cells; NSG, NOD *Prkdc*^SCID^*Il2rg^−/−^*; BRG, Balb/c *Rag2^−/−^Il2rg^−/−^*; BRGS, Balb/c *Rag2^−/−^Il2rg^−/−^ Sirpa^NOD^*; HSC, hematopoietic stem cell; FT, fetal thymus; BM, bone marrow; CAR, chimeric antigen receptor; UCB, umbilical cord blood; ATLL, T-cell leukemia/lymphoma; AML, acute myeloid leukemia; EBV, Epstein–Barr virus (37–56).

Immunodeficient mice allow great flexibility for the study of human tumor immunobiology. Human tumors can be generated in NSG, NOG, BRGS, and other strains using established tumor cell lines, after transplantation of human primary tumors or following *de novo* induction of hematological neoplasms (Figure 1). These different models provide systems that better reflect the complexity of the disease. In order to allow human tumor to engraft and grow in mice, the host immune system is generally compromised leading to tumor kinetics that may not reflect the true patient situation. As discussed earlier, human immune components can be generated *in vivo* from human HSCs or other progenitors and “supported or potentiated” later on or infused once the tumor is established. These approaches provide “mixed” systems in which human immune cells and human tumors can co-exist allowing the dissection of immune deviation as well as studying immunotherapy.

A wide range of established tumor cell lines from different origins (brain, colon, breast, melanoma, ovarian, prostate, etc.) have been engrafted in immunocompromised mice and have greatly contributed to drug development and the preclinical assessment of potential therapies. However, the gradual accumulation of genetic and phenotypic aberrations in these cells due to their long-term culture impacts the surface markers and the tumorigenicity of the malignancy (25). These limitations have set aside these models to preliminary studies addressing specific questions like the ability of a potential therapy to target a certain molecule that has been overexpressed in the cell line. In recent years, the field has been, therefore, switching toward the engraftment of patient-derived primary tumors (PDX, patient-derived xenografts) that retain the phenotypic and genetic complexity observed in clinical samples thus better predicting drug efficacy and clinical translatability (26, 27). These include tumor stromal cells and tumor-associated lymphocytes that contribute greatly to tumor growth and metastasis and, therefore, to the therapeutic response. These PDX-HIS mouse models can engraft the tumor as efficiently as the non-humanized mice, they respond to standard chemotherapeutic drugs similarly to patients and they have proven to be responsive to newly derived immune modulators.

One of the better-characterized PDX models is the AML that has contributed to the identification of leukemia stem cells (LSC) by transplanting different stem-like cell fractions and analyzing the leukemia-initiating activity of each in SCID mice (28–31). The discovery of the concept of cancer stem cell (CSC) has been a breakthrough in cancer biology due to the clinical benefits for the long-term disease-free survival. CSC presence has been identified in numerous other malignancies through transfer into immunocompromised mice and, interestingly, markers associated to CSCs have been correlated to the tumorigenic potential (32, 33). Recent improvements in HIS mouse models by the transgenic expression of certain factors, like the NSG-SGM3 expressing human SCF, GM-SCF, and IL-3, have further ameliorate the engraftment and growth of human leukemia allowing the study of the tumor initiating cells (34). The demonstration that HIS mouse models reproduce the heterogeneity and behavior of human tumors creates great expectation on the better phenotyping of these tumor-initiating cells and the identification of drugs targeting this key population (35).

One of the advantages of modeling cancer in humanized mice is that we can study the systemic environment and the contribution of nearly all the immune cells to the pathogenesis. In this regard, a NSG model of treatment-refractory B-cell leukemia revealed that infiltration of leukemia cells into the bone marrow rewires the tumor microenvironment to inhibit engulfment of antibody-targeted tumor cells. This resistance could be overcome by combination regimens involving therapeutic antibodies and chemotherapy that lead to macrophage infiltration and phagocytic activity in the bone marrow improving the efficacy of targeted therapeutics (36).

## Targeting NK Cells for Cancer Immunotherapy

To date, most immunomodulatory strategies have focused on agents or cell therapies targeting T cell immunity. In contrast, innate immune cells, such as NK cells, have been less exploited. Nevertheless, the fundamental role for these cells has been for long justified by the higher cancer incidence in individuals with defective NK function (57). Furthermore, a number of mouse models lacking or deficient for NK cell function have corroborated their importance in tumor immunosurveillance (58).

Natural killer cells exert an immediate cytotoxicity when encountering a malignant cell and they do so without a specific antigen priming but instead, by the integrated signal of an array of activating and inhibitory receptors. Among the first group, the C-type lectin-like receptors CD94/NKG2C and NKG2D and the natural cytotoxicity receptors NKp30, NKp44, and NKp46 as well as the Ig-like receptor DNAM-1 (CD266) mediate NK cell activation when they recognize tumor cells. On the other hand, polymorphic inhibitory killer cell immunoglobulin-like receptors (KIRs) with their cognate human–leukocyte–antigen (HLA) ligands as well as CD94/NKG2A with the non-classical class I molecule HLA-E as ligand provide inhibitory signaling. In addition to the contact mediated regulation of the activity, NK cells also respond to cytokines like IL-2, IL-12, IL-15, IL-18, and IL-21, as well as toll-like receptor ligands that shape their differentiation, proliferation, and activation status (59). Cytotoxicity activity is triggered through activation of the low-affinity activating receptor FcγRIIIa (CD16) that binds the Fc portion of immunoglobulin G1, which has been exploited in monoclonal antibody immunotherapies. NK cells kill virus-infected and tumor cells using a cargo of perforin and granzymes contained in cytotoxic granules and less efficiently by a mechanism dependent on FAS ligand, TNF, or TNF-related apoptosis-inducing ligand (60).

Given that NK cells in HSC-derived humanized mice express all the afore-mentioned receptors and respond similarly to the same cytokines (61, 62), these *in vivo* models represent a powerful platform to explore the pivotal role of NK cells in cancer immunosurveillance (63–65) (Lopez-Lastra et al., in revision). Additionally, environmental components such as inhibiting factors (TGF-β, IL-10, prostaglandin E2…) and immunosuppressive cells (Tregs, MDSCs) that influence the NK cell antitumor activity have also been described in HIS mice, enabling the evaluation of therapeutic strategies targeting the suppression of NK cells (66).

Although chemotherapy is still the core of the current clinical anticancer treatments, immunomodulators have now regained expectations after the revolutionary discovery of the CTLA-4 and PD-1 checkpoint inhibitors targeting T-cell activation (67). Humanized mice have proven to recapitulate the therapeutic effect of those antibodies as well as the side effects and have began to provide insights about the mechanism behind and possible strategies to improve them (68–70). The expression of these receptors on human NK cells suggests that they could also be targeted by checkpoint molecules and, therefore, contributes to the outcome of the therapy (71, 72). Indeed, mouse studies on a glioma model treated with activated NK cells preincubated with an anti-PD-1 blocking antibody showed an enhancement of the survival suggesting a role that must be explored in a human system (73).

## Adoptive Transfer of NK Cells for Cancer Therapy

The potential of NK cells as innate effectors in cancer has been studied by the adoptive transfer of *ex vivo* expanded and/or activated NK cells in immunodeficient mice. Mice treated with adoptively transferred human NK cells show NK-mediated rejection of the engrafted human tumor and further administration of cytokines, such as IL-2 and IL-15 greatly improve the NK cell pool and their cytotoxic activity against transformed cell. These observations initially made in mice laid the foundation for the autologous NK cell infusion therapies started in the 1980s for metastatic cancers (74). Preclinical assessment of cytokine regimens in other cancer models, such as the low-dose IL-2 in the spontaneous EBV-associated B-cell lymphoma in PBL-SCID mice, demonstrated reduction of the tumor load and survival prolongation (75), and preceded a number of clinical trials for both hematological and solid tumors (76–79).

The discovery that inhibitory KIRs binding to MHC-I mediate inhibition of NK cells opened a new path on NK cell immunotherapies. NOD/SCID cancer models served as a platform to confirm the higher efficacy of alloreactive NK cells for the treatment of leukemia. Contrarily to T cells, NK cell do not provoke graft-versus-host disease (GVHD) in hematopoietic stem cell transplantation (HSCT) contexts but, instead, protect the patient against it and eliminate leukemia relapse and graft rejection (80). Later on, safety and efficacy of alloreactive NK cell infusion was confirmed in the clinic by Miller and colleagues in non-HSCT settings with patients suffering from metastatic melanoma, renal cell carcinoma, Hodgkin’s lymphoma, and refractory AML (81). For many years, allogeneic NK cell infusions have been tested in the clinic with positive results and rare cases of mild toxicity (82). Strikingly, a recent pediatric clinical study has reported some patients suffering from acute GVHD after infusion of *ex vivo* expanded donor NK cells in HLA-matched HSCT (83), rising the necessity to perform more robust preclinical testing in humanized models. One strategy to do so was illustrated in a recent study performed in NSG mice, in which an alloreactive NK cell subpopulation expressing KIR2DS2 but lacking inhibitory KIR-HLA mismatch had dominant functional activation advantage to kill patient-derived glioblastoma cells (84). The regulation of the activity on infused NK cells has been classically based on HLA-KIR matching; however, other inhibitory receptors are implicated on the inhibition of NK cell cytotoxicity. A recent study in NSG mice engrafted with human HSC has shown that anti-NKG2A antibodies can stimulate human NK cell killing in AML and ALL models bypassing the need to search for NK cell alloreactive donors (85). *In vitro* experiments have also pointed to an increased NK cell-mediated lysis of lymphoma and myeloma cells with allogeneic NK cell infusion in combination with monoclonal antibodies blocking inhibitory KIRs but this effect need to be confirmed *in vivo* (86, 87).

Another strategy to increase NK cell activity without aggravating the side-effects is the expression of chimeric antigen receptors (CARs) directed against tumor antigens. Preclinical evaluation of CD20 targeting primary NK cell infusion in humanized mice has led to a clinical trial on B-lineage acute lymphoblastic leukemia currently undergoing (88). Other preclinical trials using CAR-engineered primary human NK cells are now being performed in lymphoma, leukemia, carcinomas, and neuroblastoma mouse models.

Natural killer cells are often infused in combination with immunomodulators that boost their antitumor effects or regulate their activity. CD16 receptor is targeted by many of those modulators since it mediates antibody-dependent cellular cytotoxicity (ADCC) when it recognizes an antibody on a tumor cell, leading to target cell lysis. This mechanism has been exploited by using monoclonal antibodies targeted tumor antigens thus stimulating the endogenous or adoptive NK cells. Evidences of NK cell-mediated ADCC and mild to moderate toxicity were observed in preclinical models and then confirmed for some cases in the clinical setting. Malignancies such as non-Hodgkin lymphoma with rituximab (anti-CD20), metastatic breast cancer with trastuzumab (anti-HER2) or metastatic colorectal, and squamous cell carcinoma of the head and neck have been treated with monoclonal antibodies together with NK cell infusions or in combination regimes extending the disease-free survival and overall survival of thousands of patients (89–91).

As mentioned before, CSCs are emerging as necessary targets to achieve cancer cures since current treatments eliminate the bulk of the tumor cells but rare resistant CSCs persist and lead to later tumor relapse (92). The upregulation of stress-induced antigens together with the ability of NK cells to target non-proliferating cells suggest that NK cells could effectively eliminate CSCs. Indeed, recent studies in pancreatic carcinoma-bearing NSG mice demonstrated the capacity of activated transferred NK cells to reduce intratumoral CSCs and tumor burden (93–95).

## Novel NK Cell Sources for Adoptive Therapy of Cancer

Two of the parameters to consider when evaluating the safety of NK cell products in clinical applications are the cell source and the culture conditions before the infusion. GM-CSF mobilized PBMCs, bone marrow, or umbilical cord blood (UCB) are the main sources of NK cells. With GM-CSF effects on NK function still to determine and BM being logistically difficult to obtain, UCB derived NK cells have been revealed as the best source of human material. Researchers are working on improving the expansion yield and purity as well as to enhance the activity of UCB derived NK cells before infusion in the patients. NSG mice demonstrated the capacity of these cells to migrate to BM, spleen, and liver and the inhibition of leukemia growth and prolongation of mice survival when combined with low-dose IL-15 (96). This preclinical result prompted a phase I clinical study in elderly AML patients that confirmed the safety and capacity of these cells to migrate and repopulate BM even in the absence of cytokine administration (97). This NK cell product aims at overcoming the major limitation of NK cell therapies in solid tumors, the delivery of high enough numbers of activated NK cells to the tumor site, and it is now under preclinical evaluation in the context of cervical and colorectal carcinomas (37, 98).

Alternative sources for NK cell therapy include embryonic stem cell (hESC)- or induced pluripotent stem cell (iPS)-derived NK cells, which are still under experimental development. Efficient generation of NK cells from hESC and iPS cells has been achieved, showing *in vitro* functional cytolytic activity against tumor cells, IFN-γ production, and expression of functional receptors (99). Very few reports are available regarding the *in vivo* activity of these products, with the most encouraging being in a NOD/SCID mouse model in which hESC-derived NK cells efficiently cleared a leukemia cell line tumor (100). Nevertheless, feeder-free conditions of NK cell generation need to be improved and the stability and safety of these NK cells products should be further proved in preclinical humanized models.

Finally, there is great prospect in NK cell lines as a potentially unlimited “pure” NK cell source. A clonal NK cell line NK-92 has shown the highest and most consistent cytotoxicity due to the combination of activating receptors it expresses and the absence of inhibitory KIRs (101). AML, myeloma, and melanoma are some of the numerous malignancies that have been partially eliminated from SCID mice after infusion of NK-92 (102–104). Clinical trials have further confirmed the safety and efficacy of this cell line in both solid and hematologic malignancies (105, 106). One further advantage of NK-92 is the ease of transfection with non-viral vectors allowing them to express IL-2 (required for their proliferation), thus representing a powerful “off-the-shelf” cell therapeutic (107). Additionally and inspired by the remarkable responses obtained by CAR-T cells and the early results in primary NK cells, NK-92 can be very easily transfected with a gene that expresses a tumor-CAR (108). The first preclinical tests in NSG mice have shed very optimistic results in leukemia models after CD19- or CD20-specific NK-92 infusions as well as in patient-derived glioblastoma with EGFR-specific NK-92 (109, 110). Still, these cellular therapies retain safety concerns including on-target/off-tumor effects and unregulated cytotoxicity. As such, suicide genes (including herpes-simplex-thymidine-kinase and inducible caspase-9) have been integrated into these cell products thus allowing their subsequent selective destruction (111, 112).

The latest of the NK cell therapeutic strategies was developed by Vallera and colleagues with the bi- or tri-specific killer cell engagers, BiKEs and TriKEs that are small molecules containing two or three single chain variable fragments from antibodies of different specificities (113). These are generated to bind CD16 on NK cells and one or two tumor antigens such as CD19 and CD20 (B-cell non-Hodgkin’s lymphoma) (114), CD33 or CD33 and CD123 (AML) (115), CD30 (Hodgkin’s lymphoma) (116), EGFR or EpCAM (EGFR/EpCAM overexpressing carcinomas) (117, 118), and many others. The initial preclinical evaluation in humanized mice proved very promising translational potential with results exceeding those of monoclonal antibodies, like in the case of CD16-CD19-CD20 TriKE versus rituximab, and also proved efficient for overpassing HLA-mediated inhibition in refractory AML blasts.

IL-15 is the master cytokine necessary for NK cell differentiation and survival and it is currently used in clinical trials alone or as an adjuvant for certain types of metastatic solid tumors to promote *in vivo* cell expansion and NK cell function (63, 119). Taking advantage of this, novel TriKE structures have been developed that use a human IL-15 as a modified cross-linker between the anti-CD16 and the antitumor antigen in order to promote *in vivo* NK cell proliferation. Assessment of the activity of a CD33 specific TriKE in an AML NSG model of NK cell adoptive transfer has shown *in vivo* persistence, high cytotoxic activity, and no toxicity to the construct (120). Clinical development is currently under progress and will probably obtain FDA approval in the upcoming months to be tested in patients.

## Modeling Virally Induced Human Tumors Using HIS Mice

While NK cell first identification was based on its antitumor activities, it is also a critical innate effector against pathogen invasions particularly viral infections. Human NK cells have been proven essential for the immune response against members of the herpesvirus, poxvirus, and papillomavirus families, as demonstrated by the predisposition of NK deficient individuals to suffer from these virus infections (121, 122). Remarkably, in one fifth of human cancers viral infection and oncogenesis are intimately linked. Viruses act on carcinogenesis either by directly promoting the initiation of the disease or by interacting in the immune response and/or immune evasion (123). Particularly, Epstein–Barr virus (EBV), hepatitis B virus (HBV), hepatitis C virus (HCV), human papillomavirus, human T-cell lymphotropic virus, Kaposi’s sarcoma herpesvirus, and Merkel cell polyomavirus account for the majority of tumor cases linked to viral infection. Humanized mice offer a platform to access the molecular mechanisms behind that causal role and the receptor–ligand interactions occurring at the interface “NK cell-infected cell” that could eventually have therapeutic value. The engrafted human cells occupy relevant physiological sites, where they proliferate and function, and eventually interact with oncogenic viruses that spread and replicate to other cells or organs thus recapitulating the physiological human infection. Additionally, their unique susceptibility to infection by virus with exclusive human tropism and the possibility to manipulate the timing and dose of the infection render them indispensable for better understanding the virus–tumor interplay and disease progression as well as for developing therapeutic approaches.

Epstein–Barr virus is the most common human tumor virus worldwide (more than 200,000 associated malignancies every year) and is also the cause of infectious mononucleosis. It has been extensively studied in humanized mice modeling the different protein expression patterns of the virus that lead to latent infection 0, I, II, and III as well as low level lytic replication, although only latency III has been unequivocally demonstrated (124–129). Several studies have reported specific adaptive cellular and humoral immune response to EBV in humanized mice (128, 130, 131). Furthermore, transformation of B-cell *in vivo* has been also reported and this model has disclosed one of the viral genes (EBNA3B) responsible for tumor formation (125). Preclinical studies in HIS mice have been pivotal for the development of the therapeutic vaccines that are now undergoing clinical trials, including the EBV gp350 neutralizing antibody and infusion of EBV-specific T cells (132). The involvement of NK cells in EBV infection and disease progression was demonstrated by depletion of NK cells from EBV-infected NSG mice resulting in higher EBV DNA load in the spleen, exaggerated CD8^+^ T-cell responses to the virus and an increased risk of EBV-induced lymphoproliferation (65). Current investigations try to deepen our understanding of the NK cell-mediated control of primary EBV infection in HIS mice and will likely provide insight on the NK cell subset responsible for that viral control (133).

About 80% of hepatocellular carcinomas (HCC) are due to HBV or HCV infections. There are more than 250,000 new cases of HCC and an estimated half a million deaths due to this disease annually (123, 134). We and others have developed mouse models harboring both the immune system and human hepatocytes, allowing the natural course of acute infection and also chronic hepatitis, characterized by advanced liver disease and hepatocellular carcinoma genesis (17, 135, 136). In addition to the immunodeficiency, these mice have liver defects that allow engraftment and expansion of transplanted human hepatocytes. Several immune system–liver humanized models have been developed, including BRGS-uPA (BALB/*c Rag2^−/−^Il2rg^−/−^Sirpa^NOD^uPA^tg/tg^*) (17), uPA-NOG (uPA-NOD *Prkdc*^SCID^*Il2rg^−/−^*) (135), and FRGN (*Fah^−/−^Rag2^−/−^Il2rg^−/−^* NOD) (136). These doubly humanized mice show high level of human liver chimerism and immune engraftment in primary and secondary lymphoid organs with reconstitution of myeloid and lymphoid populations at levels similar to the single HIS models. In BRGS-uPA mice, NK cells are present in spleen and liver in numbers even higher than BRGS mice and display the same NK receptor expression profile (unpublished data). Infection with HBV and HCV has been achieved in these mice and human immune responses have been detected as well as associated liver diseases that resemble the human pathology. Furthermore, both mimic the clinical response upon treatment with anti-HBs neutralizing antibodies and IFNα-2an, respectively, and prevented the leukocyte infiltration and liver fibrosis (137, 138). Any in depth analysis of the NK cell response against the virus or the role in tumorigenesis has been so far performed in these mice, other than the detection of CD56^+^ cells, to our knowledge.

## Improving the NK Cell Compartment in HIS Mice

Given the central role of NK cells in immune responses in infection, malignancy and inflammation and the great therapeutic potential they hold, it is necessary to optimize the available models for understanding their biology and preclinically evaluate new therapies.

In previous sections, we discussed about two types of HIS mice for the study of NK cell biology, those in which the human immune cells develop *in vivo* from injected hematopoietic precursors and a second category that adoptively receive mature NK cells freshly isolated or derived from an *ex vivo* expansion or activation process, a cell line or an ES or iPS cell. The later have fewer requirements in terms of niche, cell–cell interactions, and soluble growth factors that are needed for NK cell development, and instead require cytokines for their survival and homeostatic proliferation. Common cytokine receptor γ_c_ cytokines (IL-2, IL-4, IL-7, IL-9, IL-15, and IL-21) play critical roles. In particular, IL-15 is responsible for NK cell maintenance and homeostatic proliferation through IL-15Rα presentation (139, 140), while IL-2 effect *in vivo* is oriented to the activation and induction of cytotoxicity through the regulation of the peripheral NK subsets. These humanized mice serve as platforms to understand the mechanisms underlying NK survival and function and provide preclinical information for the design of new therapeutics. Furthermore, they give valuable information about the cell migration capacity and synergistic effects with other cell types or immunomodulators.

As mentioned earlier, several immunodeficient hosts (NSG, NOG, BRG, BRGS) support multilineage development of human immune cells, including low levels of NK cells. In the BRGS model (16, 141), NK cells expressed CD56 and NKp46 as well as some level of CD16 and were able to degranulate moderately after stimulation with a cancer cell line. However, in both BRGS and NSG mice, NK cell displayed defects in maturation, functionality, and heterogeneity in comparison with the human counterparts due to a deficient cytokine signaling (142). The absence of human appropriate MHC class I expression on hematopoietic or stromal cells may result in the failure to “educate” or “license” developing NK cells in HIS mice. This could explain the abundance of immature NK cells (CD56^bright^CD16^−^KIR^−^) and their functional defects. In line with this idea, recent publications showed improved NK cell licensing in a HIS models expressing diverse educating HLA alleles (143, 144). This approach may allow better definition of the mechanisms underlying human NK cell education *in vivo*.

Based on their cytokine requirements, IL-15 has been exogenously administered either alone or as a complex with IL-15Rα resulting in an extensive NK cell proliferation and accumulation of CD16^+^KIR^+^ NK cells. Also, NK cell differentiation progressed from CD56^+^ to CD56^low^CD16^+^, and finally to CD56^low^CD16^+^KIR^+^ mimicking the human model (63). On the other hand, the constitutive high expression of the high-affinity heterotrimeric IL-2 receptor complex in CD56^bright^CD16^±^ NK-cell subset and the effect of IL-2 in NK expansion and activation prompted the development of and IL-2 transgenic NOG mouse strain (145). When IL-2^Tg^NOG mice were engrafted with human HSC, CD56^+^ massively developed with a highly active phenotype including IFN-γ production and cytotoxicity against tumor cells. Interestingly, treatment of these mice with a therapeutic humanized anti-CCR4 Ab (mogamulizumab) suppressed the growth of a CCR4^+^ lymphoma, suggesting that the human NK cells in the mice exerted active Ab-dependent cellular cytotoxicity *in vivo*. These cells expressed various NK receptors, including NKp30, NKp44, NKp46, NKG2D, and CD94, as well as a diverse set of killer cell Ig-like receptor molecules at levels comparable to normal human NK cells from the peripheral blood (62). Nevertheless, there are several limitations in this model due to the supra-physiological levels of IL-2 and, therefore, the high activation status of the NK cells.

It is well known that NK homeostasis and function are regulated by the interaction with other immune cells, particularly macrophages, DCs, and T cells. In addition, soluble factors released by those cells, like NKG2D ligands, IL-2, IL-12, or IL-15, signal on NK cells leading to proliferation and activation. Based on these crosstalk events, others and we have developed humanized mice that through the enhancement of the myeloid compartment, NK cell development results improved. As it happens for other lineages, human myelopoiesis is driven by soluble factors normally present in the BM niche and periphery, which are from murine origin in HIS mice. Some of these mouse cytokines cross-react to some extent with the human cells but others, the species-specific cytokines, do not. In order to circumvent this deficiency, human cytokines have been administered to HIS mice either as recombinant proteins (63) by cytokine-encoding plasmids (146) or by insertion of the cytokines either as transgenes in the mouse genome or by knocking in the human gene to replace the mouse counterpart (147, 148). As mentioned before, transgenic models provoke supra-physiological levels of the cytokine in the periphery and in the case of pro-myeloid factors, such as TPO, IL-3, GM-CSF, or M-CSF, also lead to the exacerbated mobilization and HSC exhaustion limiting the utility of the system. Swapping mouse coding exons for M-CSF, IL-3/GM-CSF, TPO, and SIRPα with their human counterparts allowed for the creation of the MISTRG strain (149). This host expresses these human cytokines under control of mouse regulatory elements and show superior human myeloid cell engraftment. Subsequently, MISTRG HIS mice showed an increased number of functional NK cells, including higher expression of KIR, CD94, and CD161 receptors (149). Nevertheless, cellular and humoral immune responses in MISTRG HIS mice are poor and these mice develop severe anemia.

The transpresentation of IL-15 occurs mainly through the IL-15Rα expressed by DCs, so efforts have been made to increase specifically this cell population in order to increase the NK pool avoiding the overdevelopment of other myeloid subsets. In our laboratory, Flt3-deficient BRG mice (BRGF) were created and after reconstitution with human HSC, human Flt3L was administered to the mice. The result was a specific increase of all the DC subsets and the promotion of NK cell hematopoiesis, with enhanced CD94, CD16, and KIR receptor expression. The combination of this system with the expression of the *Sirpa*^NOD^ protein in the BRGSF model has led to further augmentation of NK cell numbers and also an enhanced functional competency as demonstrated by their degranulation capacity and cytokine production activity (unpublished data). This HIS model provides a unique platform to study NK cell development, crosstalk mechanisms with other immune cells, and the preclinical assessment of new immunotherapies targeting innate cells.

The combination of the protocols detailed in the previous sections for modeling human cancer or infection with the abovementioned strategies to boost the NK cells in HIS mice will raise the potential to understand how NK cell interact with malignant or infected cells. Moreover, HSC-HIS mice offer the possibility to study the tissue specific interactions, the reservoirs, the migration patterns, and the crosstalks within the immune compartment that may be important to develop combinatorial therapies that avoid metastasis, tumor relapse, and “relocation” of the viruses.

## Concluding Remarks and Future Perspectives

Therapies designed to induce or potentiate the immune response against tumors are an appealing strategy to control tumor growth and have been the object of intense research since their discovery in the 1970s. Despite representing the most promising cancer treatment since the emergence of chemotherapy, several cases of side effects or disappointing clinical results have downshifted the development of new immunotherapies. The better understanding of the tumor heterogeneity, the mechanisms of the immune response, and the interaction with the tumor microenvironment is a required step for the development of safe and effective therapies. Humanized mice have the potential to reproduce the HIS, the tumor growth and immune evasion, and the response to treatments targeting immune effector cells or immunomodulators. One of the most challenging aspects of tumor research has been to understand the variability within he same type of cancer among individuals and, therefore, the disparate responses and outcomes after therapy. Current efforts are being made to overcome these limitations by creating truly personalized HIS-PDX mouse models in which both the immune system and the tumor are derived form the same individual. These models will provide an invaluable bridge between immunotherapy discovery and the clinic, increasing the success rate of new therapies in human trials and improving the chances to beat cancer.

Natural killer cells have been for long time considered the only innate effectors of the lymphoid system but nowadays we appreciate that they belong to a larger family, the “innate lymphoid cells” (ILCs) (150). These recently described populations lack cytotoxic capacity but instead, they exert very potent cytokine production. In recent years, there has been a rapid advance in our understanding of their development, phenotypic, and functional diversity, which has been nicely reviewed elsewhere (151–155). ILCs come in three groups mirroring the cytokine and transcriptional profile of CD4^+^ helper T cells (Th1, Th2, and Th17/22). Given the myriad of cytokines they produce, ILCs have been involved in the early orchestration of immune responses against a number of pathogens, in tumor immunosurveillance and in inflammatory diseases. Recent works in mice have proven the antitumor effects of ILC1s whereas ILC3s have been found to exert both beneficial and tumor-promoting effects depending on the circumstances (156–158). The multifaceted functions of ILCs suggest new alternatives for immunotherapeutic approaches against tumors that need to be explored in *in vivo* humanized models. The improvement of human helper ILCs in HIS mice could open new avenues for harnessing innate immunity to treat cancer and inflammatory diseases.

## Author Contributions

Both authors have made substantial, direct, and intellectual contribution to the work and approved it for publication.

## Conflict of Interest Statement

JD is a stakeholder in AXENIS (founder, member of the executive board). The remaining author declares no conflict of interest.
